# International overview on juvenile-, adult- and elderly-onset rheumatoid arthritis: The age at disease onset as a fundamental determinant of clinical presentation

**DOI:** 10.1007/s10067-025-07356-5

**Published:** 2025-02-06

**Authors:** Nevin Hammam, Tahsin El-Hadidi, Khaled El-Hadidi, Ahmed Elsaman, Samah A. El-Bakry, Maha Nassr, Hanan M. El-Saadany, Doaa Mosad, Samah I. Nasef, Zahraa I. Selim, Nermeen Samy, Abdelhfeez Moshrif, Hanan Taha, Rasha M. Fawzy, Suzan S. Al-Adle, Amira M. Ibrahim, Nora Y. Elsaid, Samar Tharwat, Nada M. Gamal, Maha E. Ibrahim, Soha Senara, Rawhya El Shereef, Marwa A. Amer, Faten Ismail, Mervat I Abd Elazeem, Nouran M. Abaza, Eman F. Mohamed, Dina F. El-Essawi, Saad M. Elzokm, Samar M. Fawzy, Nahla N. Eesa, Enas A. Abdelaleem, Ahmed M. Abdalla, Hanan M. Fathi, Hatem H. El-Eishi, Safaa Sayed, Reem Hamdy A. Mohammed, Tamer A. Gheita

**Affiliations:** 1 Rheumatology Department, Faculty of Medicine, Assiut University, Assiut, Egypt; 2 Rheumatology Department, Military Academy, Agouza Rheumatology Center, Giza, Egypt; 3 Rheumatology Department, Faculty of Medicine, Cairo University, Cairo, Egypt; 4 Rheumatology Department, Faculty of Medicine, Sohag University, Sohag, Egypt; 5 Internal Medicine Department, Faculty of Medicine, Rheumatology Unit, Ain-Shams University, Cairo, Egypt; 6 Rheumatology Department, Faculty of Medicine, Fayoum University, Fayoum, Egypt; 7 Rheumatology Department, Faculty of Medicine, Tanta University, Gharbia, Egypt; 8 Rheumatology Department, Faculty of Medicine, Mansoura University, Dakahlia, Egypt; 9 Rheumatology Department, Faculty of Medicine, Suez-Canal University, Ismailia, Egypt; 10 Rheumatology Department, Faculty of Medicine, Al-Azhar University, Assiut, Egypt; 11 Internal Medicine Department, Faculty of Medicine, Rheumatology Unit, Beni-Suef University, Beni-Suef, Egypt; 12 Rheumatology Department, Faculty of Medicine, Benha University, Kalyoubia, Egypt; 13 Rheumatology Department, Faculty of Medicine, Kafr El-Skeikh University, Kafr Al Sheikh, Egypt; 14 Internal Medicine Department, Faculty of Medicine, Rheumatology and Immunology Unit, Mansoura University, Dakahlia, Egypt; 15 Rheumatology Department, Faculty of Medicine, Minia University, Minia, Egypt; 16 Rheumatology Department, Faculty of Medicine, Alexandria University, Alexandria, Egypt; 17 Rheumatology Department, Faculty of Medicine, Beni-Suef University, Beni-Suef, Egypt; 18 Rheumatology Department, Faculty of Medicine, Ain Shams University, Cairo, Egypt; 19 Internal Medicine Department, Faculty of Medicine (Girls), Rheumatology Unit, Al-Azhar University, Cairo, Egypt; 20 Internal Medicine Department, Rheumatology Unit (NCRRT), Egyptian Atomic Energy Authority (EAEA), Cairo, Egypt; 21 Rheumatology Department, Faculty of Medicine, Al-Azhar University, Damiette, Egypt; 22 Rheumatology and Rehabilitation Department, Faculty of medicine, Damietta University, Damietta, Egypt

**Keywords:** Adult-onset, Comorbidities, DAS28, Elderly-onset, Juvenile-onset, Rheumatoid arthritis

## Abstract

**Background:**

Elderly-onset rheumatoid arthritis (EORA) may have peculiar findings compared to juvenile-onset RA (JORA). The aim of the work was to present and compare the clinical characteristics of RA patients with JORA and elderly-onset EORA to a group of cases with adult-onset (AORA) and to contrast the findings worldwide.

**Methods:**

The study included 1100 adult RA patients: 209 JORA and 329 EORA, compared with 562 AORA extracted from a big data national study on 10,364 RA patients. Clinical characteristics, laboratory investigations, medications received, and co-morbidities were recorded. The disease activity index (DAS28) and health assessment questionnaire (HAQ) were estimated.

**Results:**

The JORA cases represented 19% and EORA 29.9% of the included cohort. The mean age at onset for JORA, EORA, and AORA were 15.1 ± 2.1, 64 ± 4.2, and 36.4 ± 10 years (*p* < 0.0001), and the female-male ratio was 6.2:1, 2.7:1, and 7.3:1 (*p* < 0.0001), respectively. In EORA, body mass index (28.8 ± 5.8) and frequencies of smokers (11.6%), diabetes (12.2%), hypertension (19.8%), and osteoporosis (5.2%) were significantly higher than in JORA (26.02 ± 5; 5.3%, 2.9%, 3.8%, and 1%) and AORA (27.6 ± 5.6; 3%, 8.4%, 14.9%, and 2.3%, *p* = 0.016) (*p* < 0.0001, *p* = 0.001, *p* < 0.0001, and *p* = 0.009, respectively). In JORA, oral ulcers were significantly more frequent (*p* = 0.04); in EORA, cardiovascular manifestations (*p* < 0.0001) and hypothyroidism (*p* = 0.039) were more frequent; and DAS28 (*p* = 0.01) and HAQ (*p* = 0.038) were higher. Fibromyalgia and methotrexate administration were significantly more frequent in AORA (*p* < 0.0001 and *p* = 0.04, respectively). Rheumatoid factor, anti-cyclic citrullinated peptide, and double seropositivity were significantly more frequent in EORA (*p* < 0.0001, *p* = 0.008, and *p* = 0.002, respectively).

**Conclusion:**

Comorbidities, cardiovascular manifestations, hypothyroidism, higher disease activity, and functional disability are more common in EORA patients.

**Table:** 

**Key Points** •* Juvenile-onset and elderly-onset RA patients have notable differences compared to the adult-onset cases.* •* Co-morbidities and certain manifestations, including cardiovascular disease and hypothyroidism, as well as higher disease activity and functional disability, are more common in elderly-onset patients.* •* Fibromyalgia remains more frequent in adult-onset cases.*

## Introduction

Juvenile idiopathic arthritis (JIA) comprises a group of inflammatory disorders that begin before the 18th birthday and persist for at least 6 weeks [1]. When the disease starts above 60, it is called elderly-onset RA or late-onset RA [2]. Elderly-onset RA has been shown to have peculiar findings compared to young-onset RA [3]. The spectrum of the RA phenotype in Egypt is variable across the country, with an increasing shift in the female-to-male ratio and a lower age at onset than in other countries [4]. In Japan, the age at RA onset has increased significantly over the last decade. This can be attributed to Japan’s aging population [5].

The age at which early arthritis develops has an impact on disease activity and evolution [6]. Erosion, increased disease activity, and functional disability were associated with late-onset RA. Patients with late-onset RA often receive more frequent treatment with steroids and less frequently with disease-modifying antirheumatic drugs (DMARDs), which increases their susceptibility to the development of comorbidities [7]. RA patients with late onset, fulfilling the 1987 criteria, had poor prognostic factors and higher disease activity at the time of diagnosis, which may have potential consequences for the disease course [6]. The multifaceted expression of disease activity in RA makes it challenging to provide an omni-comprehensive definition of flare [8]. The worldwide burden of JIA is intricate enough to be precisely determined. Discrepancies on classification and on the assessment of disease activity, as well as the failure to follow up during the switch of medical care from pediatric to adult rheumatology, have added to the limited understanding of the adult impact of JIA [9]. There is a clear need for a good transition from pediatric to high-quality adult rheumatology care [10].

Good RA management requires a multidisciplinary approach. The clinical condition of RA patients is recently improving due to progress in medical diagnosis and treatment that has made it possible to diminish disease activity and avoid systemic complications [11]. Even with all the enhancements in the pathogenesis and management of RA, we are still not able to prevent or cure the disease. Diagnostic delays due to a lack of access to a specialist and costly therapies are still key obstacles for many patients [12]. A significant number of patients do not reach the treatment goals [13]. Even in first-world countries, the treat-to-target principle and the goal of disease remission are often missed. Thus, RA is still the reason for disability and reduced quality of life for many patients [12].

The aim of the work was to present the clinical characteristics of juvenile-onset and elderly-onset RA compared with adult-onset RA from a large study cohort and compare the findings to those of other large cohorts worldwide. This approach aligns with global research efforts to better understand the heterogeneity of RA across different age groups and to improve patient outcomes through age-specific interventions.

## Methods

The study included 1100 RA patients fulfilling the American College of Rheumatology/European League Against Rheumatism (ACR/EULAR) classification criteria [14] extracted from a big data national study on 10,364 RA cases. The data were collected from 26 specialized rheumatology departments and centers across 22 major governorates nationwide, with the involvement of members from the Egyptian College of Rheumatology (ECR), during the period from September 2018 to December 2021. Patients with other autoimmune rheumatic diseases, current infections, or malignancies were excluded from the start. We first applied strict inclusion and exclusion criteria. We further filtered for patients with complete and high-quality data to ensure robust and reliable analyses. The sample size of 1100 was chosen to balance statistical power with practical constraints, allowing for detailed analysis and quality control while maintaining representativeness. We used random sampling to select a diverse subset reflective of the broader RA population, accounting for key variables such as age, gender, disease severity, and treatment types. Patients were divided into 3 groups in line with the known age boundaries: juvenile onset (≤ 18 years); adult onset (18–60 years); and late onset (> 60 years). Accordingly, 209 were juvenile-onset, 329 were elderly-onset, and 562 adult-onset cases.

The study was approved by the Institutional Review Board of Cairo University, Cairo, Egypt (IRB No.: 47-SReC-RCU2021). The patients provided informed consent to participate, and the study was in accordance with the 1964 Helsinki Declaration.

Patients were subjected to a full history-taking and clinical examination. The presence of co-morbidities and medications received were recorded. Fibromyalgia was diagnosed in those population by physician assessment of patient’s widespread pain symptoms not explained by the RA itself, through a thorough physical examination, and by excluding other potential causes. The laboratory investigations performed included complete blood count (CBC), erythrocyte sedimentation rate (ESR), C-reactive protein (CRP), liver and kidney function tests, rheumatoid factor (RF), anti-cyclic citrullinated peptide (anti-CCP), and antinuclear antibody (ANA). The disease activity score (DAS28) [15] and health assessment questionnaire (HAQ) [16] were assessed.

### Statistical analysis

Data was collected on a standardized data sheet and stored in an electronic database. The missing data from the various areas was considered. Statistical Package for Social Sciences (SPSS) version 25 was used. Variables were presented as frequencies and percentages or mean and standard deviation. For comparing two groups, an independent t-test for numerical variables and a chi-square test for categorical variables were used. For comparing more than two groups, one-way analysis of variance (ANOVA) for numerical variables and the chi-square test for categorical variables were used. Logistic regression analysis was considered to verify if any of the significant differences were related to the age at onset. A *P* value of < 0.05 was considered significant.

## Results

The juvenile-onset RA cases were 209, representing 19.0%, and the elderly-onset cases were 329 (29.9%) detected in the included cases. Both groups were compared to 562 (51.1%) adult-onset cases and the characteristics presented in Tables 1 and 2. Females predominated across all groups, with the highest female-to-male ratio in AORA. Comorbidities such as diabetes mellitus, hypertension, metabolic syndrome, and osteoporosis were more prevalent in EORA. Cardiovascular manifestations were significantly higher in EORA, whereas FM was more common in AORA. Disease activity, measured by DAS28 and HAQ scores, varied among groups, with EORA showing higher activity and functional impairment. Key laboratory findings include significant differences in ESR, CRP, creatinine, cholesterol, and LDL levels across the groups, with EORA RA patients showing higher ESR and CRP levels. RF and anti-CCP positivity were also more prevalent in EORA. In terms of medications, methotrexate was the most commonly used drug across all groups, with slight variations in usage rates. Steroids were frequently prescribed, particularly in adult-onset RA, while biologics were used less frequently overall.

**Table 1:** Demographic and clinical characteristics, co-morbidities and disease activity of rheumatoid arthritis according to the age at onset

Parameter n (%) or mean ± SD	All RA patients (*n* = 1100)	Onset			*p*
		Juvenile (*n* = 209)	Adult (*n* = 562)	Elderly (*n* = 329)	
Age (years)	46.9 ± 17.2	26.8 ± 8.1	42.4 ± 11.1	67.3 ± 5.1	** < 0.0001**
Gender F/M	915:185 (4.9:1)	180:29 (6.2:1)	494:68 (7.3:1)	241:88 (2.7:1)	** < 0.0001**
DD (years)	6.1 ± 6.6	11.7 ± 8.7	6.1 ± 5.9	3.6 ± 3.7	** < 0.0001**
Age at onset (years)	40.6 ± 18.8	15.1 ± 2.1	36.4 ± 10	64 ± 4.2	** < 0.0001**
BMI	27.6 ± 5.6	26.02 ± 5	27.6 ± 5.6	28.8 ± 5.8	**0.016**
Smoking	66 (6)	11 (5.3)	17 (3)	38 (11.6)	** < 0.0001**
Family hx RA	14 (1.3)	3 (1.4)	5 (0.89)	6 (1.8)	0.47
*Comorbidities*					
Diabetes mellitus	93 (8.5)	6 (2.9)	47 (8.4)	40 (12.2)	**0.001**
Hypertension	157 (14.3)	8 (3.8)	84 (14.9)	65 (19.8)	** < 0.0001**
Met Syndrome	77 (7)	7 (3.3)	32 (5.7)	38 (11.6)	**0.017**
Osteoporosis	32 (2.9)	2 (1)	13 (2.3)	17 (5.2)	**0.009**
HCV infection	10 (0.9)	0 (0)	4 (0.7)	6 (1.8)	0.07
Asthma	13 (1.2)	1 (0.5)	7 (1.2)	5 (1.5)	0.54
Hypothyroidism	32 (2.9)	1 (0.5)	17(3)	14 (4.3)	**0.039**
*Manifestations*					
RA Nodules	55 (5)	11 (5.3)	26 (4.6)	18 (5.5)	0.44
Oral ulcers	18 (1.6)	5 (2.4)	12 (2.1)	1 (0.3)	0.07
KCS/SS	138 (12.5)	18 (8.6)	84 (14.9)	36 (10.9)	0.11
Neurological	53 (4.8)	11 (5.3)	20 (3.6)	22 (6.7)	0.13
Gastrointestinal	142 (12.9)	19 (9.1)	75 (13.3)	48 (14.6)	0.16
Cardiovascular	108 (9.8)	5 (2.4)	51 (9.1)	52 (15.8)	** < 0.0001**
Chest	115 (10.5)	16 (7.7)	67 (11.9)	32 (9.7)	0.86
FMS	138 (12.5)	27(12.9)	89 (15.8)	22 (6.7)	** < 0.0001**
Renal	36 (3.3)	5 (2.4)	19 (3.4)	12 (3.6)	0.9
DAS28	4.5 ± 1.4	4.3 ± 1.6	4.4 ± 1.4	4.7 ± 1.3	**0.01**
HAQ	0.91 ± 0.61	0.98 ± 0.6	0.86 ± 0.59	1.1 ± 0.66	**0.038**

*F;* female, *M;* male, *DD;* disease duration, *BMI;* body mass index, *hx;* history, *Met Syndrome;* metabolic syndrome, *RA;* rheumatoid, *KCS;* keratoconjunctivitis sicca, *SS;* Sjögrens syndrome, *FMS;* fibromyalgia syndrome, *DAS28;* disease activity score, *HAQ;* health assessment questionnaire. Bold values are significant at *p* < 0.05

**Table 2:** Laboratory features and medications received by the rheumatoid arthritis patients according to the age at onset

Parameter *n* (%) or mean ± SD	All RA patients (*n* = 1100)	Onset			
		Juvenile (*n* = 209)	Adult (*n* = 562)	Elderly (*n* = 329)	*p*
Hemoglobin (g/dl)	11.6 ± 1.5	11.3 ± 1.2	11.6 ± 1.5	11.6 ± 1.5	0.06
TLC (× 10^3^/mm^3^)	7.1 ± 2.4	6.9 ± 2.3	7 ± 2.4	7.3 ± 2.4	0.14
Platelets (× 10^3^/mm^3^)	293.3 ± 93.8	298.8 ± 94.9	294.6 ± 90.7	288 ± 98.8	0.53
ESR (mm/1^st^hr)	46.9 ± 27.9	46.1 ± 24.5	43 ± 26.8	54.6 ± 29.9	** < 0.0001**
CRP (mg/dl)	18.9 ± 23.2	21.6 ± 29.9	15.7 ± 18.6	23.9 ± 26.3	**0.001**
ALT (IU/l)	23.7 ± 13.9	21.3 ± 15.1	24.8 ± 14.9	23 ± 10.9	**0.048**
Creatinine (mg/dl)	0.8 ± 0.34	0.7 ± 0.24	0.79 ± 0.38	0.89 ± 0.3	** < 0.0001**
Cholesterol (mg/dl)	205.6 ± 59.2	185.5 ± 52.9	216.3 ± 59.2	191.6 ± 57.4	**0.002**
Triglycerides (mg/dl)	124.1 ± 44.9	114.7 ± 43.3	127.2 ± 41	122.5 ± 54.5	0.34
HDL (mg/dl)	54.1 ± 25.6	60.9 ± 33.9	54.9 ± 25.8	47.04 ± 15.04	0.052
LDL (mg/dl)	111.3 ± 38	95.01 ± 32.6	111.1 ± 29.4	123.8 ± 54.2	**0.003**
SUA (mg/dl)	4.9 ± 1.6	4.3 ± 2.03	5.02 ± 1.3	4.9 ± 1.7	0.14
RF (*n* = 850)	621 (73.1)	100/144 (69.4)	315/453 (69.5)	206/253 (81.4)	** < 0.0001**
Anti-CCP (*n* = 600)	403 (67.2)	45/83 (54.2)	246/362 (68)	112/154 (72.7)	**0.008**
DSP (*n* = 577)	313 (54.2)	34/79 (43)	186/353 (52.7)	93/145 (64.1)	**0.002**
ANA (*n* = 315)	48 (15.2)	7/52 (13.5)	30/190 (15.8)	11/73 (15.1)	0.92
Anti-dsDNA (*n* = 92)	1 (1.1)	0 (0)	0 (0)	1/31 (3.2)	-
*Medications*					
Steroids	641 (58.3)	106 (50.7)	366 (65.1)	169 (51.4)	0.07
Methotrexate	744 (67.6)	141 (67.5)	386 (68.7)	217 (66)	**0.04**
Hydroxychloroquine	534 (48.5)	88 (42.1)	306 (54.4)	140 (42.6)	0.34
Leflunomide	372 (33.8)	69 (33)	211 (37.5)	92 (28)	0.10
Sulfasalazine	35 (3.2)	12 (5.7)	11 (2)	12 (3.6)	0.17
Azathioprine	14 (1.3)	1 (0.48)	7 (1.2)	6 (1.8)	0.58
Biologics	75 (6.8)	15 (7.2)	40 (7.1)	20 (6.1)	0.28

*TLC;* total leucocytic count, *ESR;* erythrocyte sedimentation rate, *CRP;* C-reactive protein, *ALT;* alanine transaminase, *HDL;* high density lipoprotein, *LDL;* low density lipoprotein, *SUA;* serum uric acid, *RF;* rheumatoid factor, *anti-CCP;* anti-cyclic citrullinated peptide, *DSP;* double seropositivity *ANA;* anti-nuclear antibody. Bold values are significant at *p* < 0.05

On regression analysis, the age at onset was associated with CVS manifestations; use of MTX, RF, anti-CCP, and double seropositivity remained significant at *p* = 0.016, *p* < 0.0001, *p* = 0.002, *p* < 0.0001, and *p* = 0.001, respectively.

Tables 3, 4, and 5 provide a comparative analysis of RA features across different age groups and various countries. The tables highlight demographic characteristics, comorbidities, seropositivity rates, disease activity, and medication usage.

**Table 3:** World-wide features of elderly-onset rheumatoid arthritis

Variable	USA [22]	Colombia [23]	Singapore [24]	China [25]	Japan [26]	Pakistan [3]	Poland [27]	Intl [28]	Egypt *this work*
Number	40	104	178	212	46	32	63	705	329
Mean age (y)	−	72.8	−	72	77	65.5	73.6	−	67.3
Onset age (y)	67.1	70.4	66.7	68	75.6	−	64	73.3	64
F/M	1.7:1	1.8:1	3.3:1	2.1:1	3.6:1	0.7:1	2.9:1	1.9:1	2.7:1
*Comorbidity*									
Hypertension	−	−	52.8%	44.8%	50%	−	−	−	19.8%
Diabetes	−	−	18.5%	22.2%	21.7%	−	−	−	12.2%
Osteoporosis	−	−	33.7%	−	-	−	−	−	5.2%
RF	75%	49.5%	71.1%	85.3%	73.9%	87.5%	90.5%	67.5%	81.4%
Anti-CCP	69%	54.5%	−	80.4%	70%	90.6%	90.5%	67.5%	72.7%
DSP	−	−	−	−	−	−	90%	−	64.1%
Mean DAS28	−	6	−	4.6	4.3	−	4.4	3.7	4.7
Mean HAQ	−	1.16	0.65	−	−	−	−	1.2	1.1
*Medications*:									
Steroids	68%	−	93.8%	23.6%	100%	81%	77.8%	30.1%	51.4%
MTX	68%	−	57.3%	21.2%	58.7%	53.1%	85.7%	53.9%	66%
HCQ	38%	−	29.2%	18.4%	−	19%	9.5%	30%	42.6%
LFN	−	−	0.6%	40.6%	−	34.4%	−	−	28%
Biologics	100%	−	0%	9.4%	26.1%	12.5%	9.5%	−	6.1%

*RA;* rheumatoid arthritis, *RF;* rheumatoid factor, *anti-CCP;* anti-cyclic citrullinated peptide, *DSP;* double seropositivity, *DAS28;* disease activity score, *HAQ;* health assessment questionnaire, *MTX;* methotrexate, *HCQ;* hydroxychloroquine, *LFN;* leflunomide

**Table 4:** World-wide features of adult-onset rheumatoid arthritis patients

Variable	USA [22]	Colombia [23]	Singapore [24]	China [25]	Pakistan [3]	Poland [27]	Egypt *this work*
Number	116	96	1028	904	187	50	562
Mean age (y)	-	46.1	-	53	40.7	41.5	42.4
Onset age (y)	43.7	43.3	41.4	43	−	24	36.4
F/M	2.7:1	3:1	5.8:1	4.4:1	3.7:1	−	7.3:1
*Comorbidity*							
Hypertension	−	−	27.5%	22%	−	−	14.9%
Diabetes	−	−	8.8%	9.8%	−	−	8.4%
Osteoporosis	−	−	14.3%	−	−	−	2.3%
RF	68%	62.8%	78.6%	86.2%	88%	84%	69.5%
Anti-CCP	58%	−	−	81.9%	82%	86%	68%
DSP	−	−	−	−	−	78%	52.7%
Mean DAS28	−	6.7	−	4.5	−	3.2	4.4
Mean HAQ	−	1.15	0.55	−	−	−	0.86
*Medications*:							
Steroids	57%	−	91.3%	22.3%	18%	94%	65.1%
MTX	65%	−	74.7%	28.9%	50.3%	52%	68.7%
HCQ	30%	−	42.7%	34.3%	25%	12%	54.4%
LFN	−	−	4.9%	38.4%	25%	−	37.5%
Biologics	100%	−	0.6%	11.9%	14%	68%	7.1%

*RA;* rheumatoid arthritis, *RF;* rheumatoid factor, *anti-CCP;* anti-cyclic citrullinated peptide, *DSP;* double seropositivity, *DAS28;* disease activity score, *HAQ;* health assessment questionnaire, *MTX;* methotrexate, *HCQ;* hydroxychloroquine, *LFN;* leflunomide

**Table 5:** Worldwide features of juvenile-onset rheumatoid arthritis

Variable	USA [31]	Brazil [21]	UK [10]	Portugal [9]	Egypt *this work*
Number	45	27	246	426	209
Mean age (y)	27.4	28.1	35.4	34.1	26.8
Onset age (y)	6.8	10	7.1	9.9	15.1
F/M	8:1	2.4:1	2.5:1	2.1:1	6.2:1
*Comorbidity*					
Hypertension	−	−	−	−	3.8%
Diabetes	−	−	−	−	2.9%
Osteoporosis	−	−	25.2%	−	1%
RF	29%	−	15.1%	27.5%	69.4%
Anti-CCP	28.6%	−	−	30.8%	54.2%
DSP	−	−	−	−	43%
ANA	−	−	−	30.7%	13.5%
Mean DAS28	−	−	−	−	4.3
Mean HAQ	−	1.5	−	0.5 ± 0.7	0.98
*Medications*:					
Steroids	−	−	58.9%	25.8%	50.7%
MTX	66.7%	−	40.7%	−	67.5%
HCQ	13.3%	−	20.9%	−	42.1%
LFN	6.7%	−	−	−	33%
Biologics	35.6%	66.6%	−	7.8%	7.2%

*RA;* rheumatoid arthritis, *RF;* rheumatoid factor, *anti-CCP;* anti-cyclic citrullinated peptide, *DSP;* double seropositivity, *DAS28;* disease activity score, *HAQ;* health assessment questionnaire, *MTX;* methotrexate, *HCQ;* hydroxychloroquine, *LFN;* leflunomide

## Discussion

The study provides significant insights into the clinical characteristics of RA across different age groups, particularly comparing JORA, EORA, and AORA. Key findings include the higher prevalence of comorbidities such as diabetes, hypertension, and osteoporosis in EORA patients, along with increased body mass index and smoking rates. EORA patients also exhibited higher disease activity (DAS28) and greater functional disability (HAQ) compared with JORA and AORA patients. Notably, EORA patients had higher rates of seropositivity for rheumatoid factor, anti-cyclic citrullinated peptide antibodies, and double seropositivity, indicating a more pronounced autoimmune response. Age-specific manifestations were observed, with JORA patients having more oral ulcers and EORA patients experiencing more cardiovascular manifestations and hypothyroidism. AORA patients showed a higher prevalence of FMS and more frequent use of MTX. The novelty of this study lies in its comprehensive comparison of RA characteristics across different age groups within a large national cohort, highlighting the distinct clinical profiles and management needs of JORA, EORA, and AORA patients. These findings underscore the importance of age-specific approaches in the diagnosis and treatment of RA, particularly emphasizing the unique challenges associated with elderly-onset disease.

Age at disease onset is a potential determinant of disease activity, clinical course, and outcome [17]. Many patients with JIA are followed into adulthood, evolving differently [9], and with the aging of the general population in many countries, the onset of RA at an older age is becoming more common [18]. Even though elderly-onset RA is considered after the age of 60, characteristics were more evident after 65 years [19]. Aging has a possible effect on the clinical presentation of hyperthyroidism, systemic lupus erythematosus, and elderly-onset RA [20]. The management of elderly-onset RA is challenging due to progressive functional disability, increased comorbidities, and high drug-related risks [19]. These differences are significant not only in highlighting the importance of the aging process on the immune system but also because they have medical and therapeutic implications [20]. However, RA can no longer be considered a disease of elderly people, as the majority of RA patients are middle-aged; notably, early diagnosis is a key reason [17]. Adult Brazilian patients with JIA had a later disease onset and required more biologic treatment than children [21]. Differentiating the various characteristics of juvenile-onset, adult-onset, and elderly-onset RA patients from different countries [3, 22–28] is compared with the current findings in Tables 3–5.

Juvenile idiopathic arthritis (JIA) persisting into adulthood is associated with articular damage, increased disability, and mortality [29]. Adults with JIA usually have significant levels of disability due to ongoing activity for a long time [10]. In the work of June et al. [29], juvenile-onset RA patients were 9:1 F/M, 29% positive RF, 28.6% positive anti-CCP, 58.1% positive ANA, HAQ 0.43, 4.4% hypertensive, and MTX was received by 68.9%.

Approximately half of all young adults with JIA have ongoing active disease, and over one-third experience detectable degrees of disability and organ damage. These patients also show a distinctive pattern of growth disturbances [30]. The gap in clinical knowledge is behind the remarkable frequency of adult rheumatologists assigning a diagnosis of RA to JIA patients immediately after transition from pediatric care, yet such cases should be considered a unique population of RA [31]. Most JIA patients continue their management at adult rheumatology centers to receive adequate long-term treatment; however, almost 50% of the transfer processes fail, with an increased risk of unfavorable outcomes. Obstacles to the success of transitional care for JIA patients include the absence of explicit assessment tools for disease activity and the lack of particular treatment guidelines for JIA adult patients [32].

The gender distribution showed disparity, with a lower F/M ratio in the elderly-onset patients. Elderly-onset RA had dissimilar disease characteristics compared to those with young-onset RA. The gender distribution is more equal in elderly-onset RA [33]. Given the obvious phenotypical differences of age, gender, and family history, more refractory disease suggest that elderly-onset RA is perhaps genetically different [3]. Elderly-onset RA is a unique subset characterized by poor prognosis at multiple levels and is no longer considered a benign form of disease [34]. The immunomodulatory actions of sex hormones have been demonstrated [35]. The proportion of women and men is comparable in elderly-onset RA [27]. Late-onset RA is characterized by an equal gender distribution [17].

In this work, elderly-onset RA had a higher frequency of first-degree relatives, smoking, BMI, and comorbidities. Diabetes, hypertension, and MetS were significantly increased in elderly-onset patients. Furthermore, it is expected that dyslipidemia, in the form of cholesterol and LDL, was significantly higher.

It is unsurprising to find that the frequency of osteoporosis was higher in the elderly-onset group, especially as they were of higher age during the study. Autoimmune inflammatory processes, which lead to a systemic upregulation of inflammatory and osteoclastogenic cytokines, the production of autoantibodies, and Th cell senescence with a presumed disability to control the systemic immune system’s and osteoclastogenic status, may play important roles in the pathophysiology of osteoporosis in RA [36].

The prevalence of features of geriatric syndromes was higher in elderly RA patients than in healthy elderly and young RA patients [37]. In this work, other associated diseases, including HCV infection, bronchial asthma, and hypothyroidism, were more common in EORA patients.

It is expected that cardiovascular manifestations were significantly increased in elderly-onset patients, especially with the significantly increased risk factors. In fact, the presence of RF or ACPA is associated with cardiovascular disease (CVD) and mortality among RA patients with disease onset before 65 years. Age stratification may improve the evaluation of risk for CVD and mortality in early RA [38]. Gastrointestinal manifestations were also more present in EORA cases. The various clinical presentations may cause difficulties in the diagnosis of EORA [33]. There are significant changes in the gut microbiota composition of RA patients due to environmental factors and changes in diet and nutrition [39]. In this work, neuropsychiatric manifestations tended to be higher in the elderly-onset group of patients. RA has been linked to neuropsychiatric comorbidities and an increasing risk of neurodegenerative diseases in life. Furthermore, immune dysfunction stimulates the peripheral and central neuronal systems that regulate innate and adaptive immunity and the neuro-immune crosstalk process [40].

Disease activity and functional disability were significantly increased in elderly-onset patients. It is also surprising to see the elevated ESR and CRP in this vulnerable group. An international study on 7912 RA patients revealed that 8.9% had an onset age of over 65 years, with a higher proportion of males, a higher seronegative rate, and a slightly different treatment approach compared with patients with a younger onset age. However, there were no significant changes in treatment strategies or the response to treatment, as measured by DAS and HAQ [28]. The prevalence of some features of geriatric syndromes was higher in elderly RA patients than in healthy elderly and young RA patients [37]. The clinical course of elderly-onset RA is characterized by high disease activity [27]. Questions on how to predict the aggressiveness of the disease course and how changes in the cellular immune system are associated with disease activity in patients of younger and older ages exist [17]. Elderly patients with RA are frequently associated with higher disease activity and impaired physical function, although they show intolerance for conventional synthetic disease-modifying antirheumatic drugs (csDMARDs), such as methotrexate, because of their comorbidities. However, the present treatment recommendation based on randomized controlled trials is not distinguished by age or comorbidities. Both TNFi and non-TNFi agents may not strongly influence the risk of adverse events such as cardiovascular events and malignancy in elderly patients. Regarding JAKi, the efficacy appears to be similar, although the safety (particularly for serious infections, including herpes zoster) may be attenuated by aging [41]. As cases in remission could have been lost for follow-up, the patients in this study may over-represent more severe cases.

The autoantibodies RF, anti-CCP, and double seropositivity were significantly higher in elderly-onset RA patients. The causes of different courses of RA in elderly people are not yet known, as on the contrary, the frequencies of positive anti-CCP, erosion score, or serum CRP levels did not differ between late-onset and young-onset RA patients [17], while a lower frequency of RF positivity was reported in elderly-onset RA [17, 33].

Methotrexate, steroids, and HCQ were more frequently used by adult-onset “reproductive” patients, as MTX, LFN, and HCQ usage were lower in the elderly-onset group. Less disease-modifying antirheumatic drug usage was reported as distinguishing elderly-onset from young-onset RA patients [26]. The treatment strategy of patients with young-onset RA should follow the same principles of aggressive early intervention and treat-to-target, aiming at stringent disease control and structural damage prevention. Nevertheless, this is often not the case in clinical practice [34], and increased comorbidities, limited communication, frailty, and poly-medication are multifactorial reasons [42]. Moreover, rheumatologists tend to treat elderly-onset RA less aggressively [43]. A shift to a more determined approach to elderly-onset RA is warranted to improve the potentially unfavorable prognosis [34].

A significant difference in biologics use among elderly-onset RA between centuries was identified. In the current study, 6.1% of elderly-onset RA used biologics for the management of RA. Richter et al. reported biologics use in all elderly-onset RA patients included in the study, while Tan et al. demonstrated no biologics use among elderly-onset RA in Asian cohort. The difference in biologics use in rheumatology between countries is well-known and attributed to the level of access patients have due to factors like healthcare system structure, drug reimbursement policies, and affordability, leading to variations in the types and frequency of biologics prescribed depending on the country; essentially, some countries have wider availability and usage of biologics compared with others due to economic constraints and insurance coverage.

Biologic therapy was used more with the juvenile-onset patients. In line, adults with JIA are often treated with biologic therapies, but the choice of the first biologic agent is frequently inconsistent due to the lack of specific guidelines [32]. JIA can be a lifelong disorder. With early and more widespread use of biological and other innovative therapies, however, outcomes for patients with JIA should improve further [30]. Depicting the disparity between young-onset and elderly-onset RA should enhance therapeutic decisions [20].

The study has several limitations that should be considered when interpreting its findings. First, the cross-sectional design limits the ability to establish causal relationships between variables and outcomes, as it only provides a snapshot of the data at a single point in time. Second, the study does not account for potential variations in medication adherence, which could significantly influence disease activity and outcomes. Third, the lack of long-term follow-up data restricts insights into the progression and long-term outcomes of RA across different age groups. Additionally, the findings may not be generalizable to other geographic or ethnic populations due to regional differences in RA presentation and management practices. Forth, we acknowledge that some degree of selection bias may still exist. Finally, the prevalence of comorbidities might be underreported or influenced by the diagnostic capabilities and practices of the healthcare system, potentially affecting the accuracy of the results. Addressing these limitations in future research would provide a more comprehensive understanding of the clinical characteristics and management of RA across different age groups.

In conclusion, the juvenile-onset and elderly-onset RA patients show notable differences compared with the adult-onset cases. Comorbidities and certain manifestations, including cardiovascular disease and hypothyroidism, as well as higher disease activity and functional disability, are more common in elderly-onset patients, and special attention is warranted for this particular group. Fibromyalgia remains more frequent in adult-onset cases. There was a tendency to use biologic therapy more frequently in juvenile-onset cases. There is a clear need for a good transition from pediatric to high-quality adult rheumatology care in order to enable real progress in terms of quality of life, work practice, satisfaction with care, and long-term outcomes.

## Data Availability

The datasets used and/or analyzed during the current study are available from the corresponding author on reasonable request.
